# Transfers from intensive care unit to hospital ward: a multicentre textual analysis of physician progress notes

**DOI:** 10.1186/s13054-018-1941-0

**Published:** 2018-01-28

**Authors:** Kyla N. Brown, Jeanna Parsons Leigh, Hasham Kamran, Sean M. Bagshaw, Rob A. Fowler, Peter M. Dodek, Alexis F. Turgeon, Alan J. Forster, Francois Lamontagne, Andrea Soo, Henry T. Stelfox

**Affiliations:** 1 Calgary, Canada; 2 Edmonton, Canada; 3 Toronto, Canada; 4 Vancouver, Canada; 5 Québec, Canada; 6 Ottawa, Canada; 7 Sherbrooke, Canada

**Keywords:** Provider communication, Documentation, Patient transfer, Intensive care unit, Progress notes, Hospital ward

## Abstract

**Background:**

Little is known about documentation during transitions of patient care between clinical specialties. Therefore, we examined the focus, structure and purpose of physician progress notes for patients transferred from the intensive care unit (ICU) to hospital ward to identify opportunities to improve communication breaks.

**Methods:**

This was a prospective cohort study in ten Canadian hospitals. We analyzed physician progress notes for consenting adult patients transferred from a medical-surgical ICU to hospital ward. The number, length, legibility and content of notes was counted and compared across care settings using mixed-effects linear regression models accounting for clustering within hospitals. Qualitative content analyses were conducted on a stratified random sample of 32 patients.

**Results:**

A total of 447 patient medical records that included 7052 progress notes (mean 2.1 notes/patient/day 95% CI 1.9–2.3) were analyzed. Notes written by the ICU team were significantly longer than notes written by the ward team (mean lines of text 21 vs. 15, *p* < 0.001). There was a discrepancy between documentation of patient issues in the last ICU and first ward notes; mean agreement of patient issues was 42% [95% CI 31–53%]. Qualitative analyses identified eight themes related to focus (central point – e.g., problem list), structure (organization, – e.g., note-taking style), and purpose (intention – e.g., documentation of patient course) of the notes that varied across clinical specialties and physician seniority.

**Conclusions:**

Important gaps and variations in written documentation during transitions of patient care between ICU and hospital ward physicians are common, and include discrepancies in documentation of patient information.

**Electronic supplementary material:**

The online version of this article (10.1186/s13054-018-1941-0) contains supplementary material, which is available to authorized users.

## Background

Transitions of patient care are vulnerable periods in healthcare delivery that expose patients to potential breakdowns in communication [1–3], medical errors [4], and adverse events [5, 6]. The transfer of a patient from the intensive care unit (ICU) to a hospital ward represents an example of a common high-risk inter-specialty transition of care where patients with complex life-threatening problems transition from the care of a critical care medicine physician to a medical, surgical or primary care physician. Ineffective handoffs can lead to approximately 10% of adverse events in the ICU [7]. Efforts to improve transitions of care have focused on patient transfers during end-of-shift [2, 8, 9] or end-of-service [10–12] handoffs. However, there may be differences in culture and clinical focus between providers in different service areas of a hospital [13–15], such as the transfer of a patient from the intensive care unit (ICU) to a hospital ward.

A report from the Canadian Institute for Health Information found that unintended harm occurs in one of every 18 hospitalizations [16]. Communication issues are a root cause of such adverse events [17]. Effective transitions of patient care, which have been previously described as including continuous communication [10] and coordination [18] of patient needs across the healthcare continuum, contribute to improved patient care [19, 20], safety [10, 21], and experience [21]. The medical record is central to this process because it is the repository for documenting events [22, 23], current issues [24], and services provided [25]. It is a source of durable information that healthcare providers and in some institutions, patients [26, 27] can access when making clinical decisions [24, 28]. Physician progress notes are a core component of the medical record [22, 29] and efforts to optimize documentation have included the development and implementation of standardized forms [22, 30], such as the subjective, objective, assessment, and plan (SOAP) model [31] for paper-based records, or templates for electronic health records (EHRs) [32]. However, there is limited evidence about how information should be displayed in the medical record [33, 34], and little is known about documentation during transitions of patient care between different medical specialty teams [35, 36]. Moreover, a systematic review of the literature on patient handoff tools reported little standardization across handoff practices, including the structural organization of tools currently in use [37], highlighting the need for an additional research focus on the contextual nuances of handoffs and their effect on patient-related outcomes [37]. Our goal was to contribute to this gap in the literature by describing current text-based communication practices during transfers of patient care from ICU to a hospital ward, and identifying opportunities to improve communication and ultimately patient safety.

## Methods

### Study design

This study was part of a multicentre prospective cohort study [38] describing transfers of care for adult patients who were discharged from medical-surgical ICUs to hospital wards between July 2014 and January 2016 in ten hospitals across seven Canadian cities. The study hospitals were selected to ensure a diversity of institutions including tertiary and community care, teaching, non-teaching, size of hospitals, English- and French-language hospitals, and geographic representation. The Health Research Ethics Board at the coordinating center (University of Calgary REB13-0021) and at each of the study hospitals approved the study. We targeted enrollment of 50 consecutive consenting patients identified as ready for transfer from the ICU to a hospital ward at each study site (n = 500), but ended recruitment at 447 patients due to time and resource limitations. Detailed descriptions of the protocol for this study and the textual analyses used in this study have been published previously [39, 40].

### Data collection

Paper medical records were collected for all enrolled patients (see Additional file 1). Physician progress notes within each record were collected for up to ten consecutive calendar days depending on the length of hospital stay: up to 2 days in ICU before transfer, the day of transfer, and up to 7 days after transfer to the accepting hospital ward. Notes were photocopied, de-identified, and assigned a unique identifier for analysis.

### Analytic approach

Notes were analyzed to describe communication around the time of transfer from the ICU to the accepting hospital ward. Two unique coding systems—descriptive and content analyses [39]—were developed to capture quantitative and qualitative concepts of the notes, respectively. Through a preliminary analysis using open coding methodology [41], overarching themes emerged to describe: focus (i.e., central point), structure (i.e., organization) [39], and purpose (i.e., intention) of text-based communication in physician progress notes. A codebook (Appendix A) was developed by two reviewers (KB, JPL) [39] to systematically identify granular themes for quantitative and qualitative concepts.

### Descriptive analyses

Descriptive analyses included tabulation of basic descriptors (e.g., date), readability (e.g., legibility), the type of information (e.g., patient history) and communication documented (e.g., provider-provider, provider-family). Two reviewers (KB, HK) independently coded all medical records to capture these quantitative items [39]. Inter-rater reliability for nominal quantitative codes were evaluated on a random sample of 37 medical records (median kappa score 0.95, 95% confidence interval [CI] 0.96–0.99). Notes written during the ICU stay were compared to those written during the ward stay and between medical and surgical patients using mixed-effects linear regression models accounting for patients clustered within hospitals. Results were summarized using means or mean percentages with 95% CIs. Analyses were done using Stata version 14 (StataCorp LP, College Station, TX, USA).

### Content analyses

To provide a more in-depth description of charting across institutions, content analyses were applied to a purposive, random sample of medical records from each of the eight study hospitals where English was the language used in the medical record (n = 2 surgical patients and n = 2 medical patients per hospital, totaling n = 32 medical records, see Additional file 2) with the overall goal of capturing similarities and differences in the focus, structure, and purpose of each progress note. A description of the inductive approach that led us to focus on these overarching trends in the data has been published elsewhere [39]. While our team was prepared to increase the number of charts in our sample if necessary, ongoing analysis revealed distinct recurring patterns in the data, with no new themes emerging prior to the conclusion of our analysis on the initial sample of 32. Emphasis was placed on variation across: (1) medical versus surgical specialties, (2) hospitals, (3) levels of physician seniority, and (4) temporality. Moreover, we examined the consistency of medical issues (i.e., problem list) documented in the last note written by the ICU physician team, and the first note written by the physician team assuming care on the hospital ward. The number of common issues between the two notes was divided by the total number of unique issues in both notes to obtain the measure of acceptance.

## Results

### Findings from quantitative descriptive analyses

#### Documentation, readability, and mode of communication

**Fig. 1:**
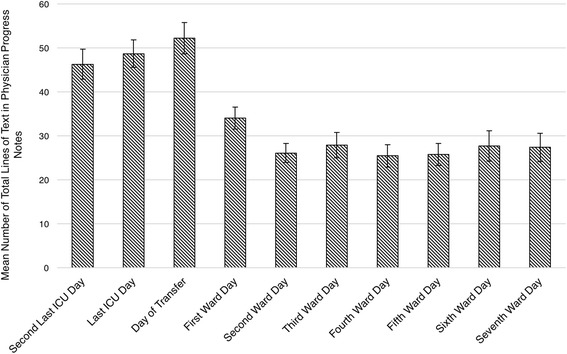
Number of total lines of text in physician progress notes during transition of patient care. The *bars* represent the mean number of total lines of text in physician progress notes according to each day within the 10-day time period. 95% confidence intervals are displayed on each bar

From the 447 medical records collected (370 written in English), a total of 7052 individual physician progress notes were identified and analyzed (Table 1). There was a mean of 2.1 notes/patient/day [95% CI 1.9–2.3] during the 10-day period. Most notes were handwritten (97% [95% CI 89–100%]). None of the included hospitals employed an integrated electronic health record (EHR) for ICU, transition, and ward notes. One ICU (Hospital H) used an electronic Word document template to type progress notes that were subsequently printed on a separate piece of paper for placement in the medical record. Of all notes (handwritten and typed) per patient, 87% [95% CI 78–96%] were legible (Appendix A), 95% [95% CI 92–98%] included a date, 49% [95% CI 40–58%] included a time, and 48% [95% CI 34–62%] included the identification of the writer (e.g., full name and/or pager number). Notes written by ICU team members were more frequent (mean/patient/day 2.4 vs. 1.8, *p* < 0.001) and longer (mean number of lines of text 21 vs. 15, *p* < 0.001) than those written by ward team members (Fig. 1). Most lines of text in notes described patient data (i.e., physician’s clinical assessment and test results), while fewer lines were dedicated to describing the patient’s history (i.e., purpose of hospitalization, medical history) or clinical plan. A minority of progress notes documented communication between providers, providers-patients or -patient families. Similar documentation patterns were observed in the notes of patients who had medical or surgical diagnoses with a few exceptions. Compared to surgical patients, notes for medical patients were longer (mean number of lines of text 19 vs. 15 *p* < 0.001), and had more text about the clinical plan (32% vs. 30%, *p* = 0.009) and about communication between providers or between providers and patients/family members (10% vs. 7%, *p* = 0.003) (see Additional files 3 and 4).

**Table 1:** Quantitative descriptive analysis of physician progress notes

Measures	Total (n = 7052)	ICU stay (n = 1966)	Transfer day (n = 1207)	Ward stay (n = 3879)	*p* value^a^
Mean number of notes per day (per patient)	2.1 [1.9–2.3]	2.4 [2.2–2.6]	2.8 [2.6–3.0]	1.8 [1.6–2.0]	*p* < 0.001
Handwritten	97% [92–100%]	93% [87–100%]	93% [87–100%]	100% [94–100%]	*p* < 0.001
Legible	87% [78–96%]	86% [77–95%]	87% [78–96%]	87% [78–96%]	*p* = 0.458
Date included	95% [92–98%]	95% [92–98%]	96% [93–99%]	95% [92–98%]	*p* = 0.778
Time-stamped	49% [40–58%]	51% [41–61%]	54% [44–64%]	46% [36–56%]	*p* = 0.006
Signature/name	48% [34–62%]	48% [34–62%]	48% [34–62%]	47% [33–60%]	*p* = 0.630
Mean number of lines per note (per patient)	17.6 [15.9–19.3]	21.1 [19.1–23.1]	19.6 [17.6–21.6]	15.0 [13.1–17.0]	*p* < 0.001
ᅟPatient history^b^	10% [7--13%]	11% [9–14%]	11% [8–13%]	7% [5–10%]	*p* < 0.001
ᅟPatient data^b^	59% [54–64%]	57% [52–62%]	58% [53–63%]	61% [56–66%]	*p* < 0.001
ᅟPatient care plan^b^	31% [26–37%]	32% [26–37%]	32% [26–38%]	31% [26–37%]	*p* = 0.701
Communication^c^	9% [7–10%]	7% [5–9%]	8% [6–10%]	9% [7–11%]	*p* = 0.071
ᅟProvider-provider^d^	56% [50–62%]	64% [56–73%]	62% [52–72%]	51% [44–59%]	*p* = 0.012
ᅟProvider-family^d^	23% [16–29%]	23% [15–31%]	22% [12–31%]	22% [15–29%]	*p* = 0.851
ᅟProvider-patient^d^	38% [33–43%]	29% [20–38%]	36% [26–46%]	43% [35–50%]	*p* = 0.007

Data are presented as mean proportions (95% confidence intervals) unless otherwise indicated. Data include notes written by ICU and ward physicians categorized according to patient location during the 10-day period. Indented variables are presented as distributions

^a^*P* values represent the comparison between the ICU stay and ward stay for each variable

^b^Patient history (e.g., symptoms), data (e.g., laboratory results), and care plan (e.g., treatments prescribed) are presented as mean proportions (%) of the total number of lines for each note

^c^Documentation of any communication between providers, between providers and family members, and between providers and patients

^d^Provider-provider, -family, -patient communication presented as mean proportions (%) of the total documented communication that is not mutually exclusive

### Themes identified from qualitative content analyses (n = 32)

Of the 32 medical records included in the content analyses, the majority of notes (73%, 95% CI 54–91%) with an identifiable writer were written by trainees (e.g., residents, fellows).

#### Focus

Physician progress notes were largely comprised of three main areas of focus: documentation of a patient’s ongoing medical issues (i.e., problem list), physician subjective interpretation of a patient’s status and future course of treatment (i.e., clinical impression), and a summary of events that occurred within a specific time frame (e.g., overnight).

#### Problem list

Discrepancies in the documentation of patient’s problems emerged as a key observation during the transition of care from ICU to hospital ward. Notes written by staff physicians were more likely to document one or two main issues of the patient. Trainee notes were more likely to include a numbered problem list and corresponding clinical plan (Additional file 5: Table S5). ICU and medical teaching unit notes regularly included a head-to-toe assessment. Surgical specialty notes frequently included information specific to an injury or disease.

The mean number of issues reported in the last ICU physician progress note was not significantly different from the number documented in the first ward note (3.4 vs. 2.9, *p* = 0.461). However, the mean agreement of issues listed in the last ICU note and the first ward note was only 42% [95% CI 31–53%] (i.e., there was discrepancy of information in both notes). Similar findings were observed for patients who had medical (42%) and surgical (42%) diagnoses, but varied substantially between hospitals (range 20–65%).

#### Clinical impression

ICU staff physician notes were more likely to document provider-patient and provider-family communication than trainee notes. Often, ICU and hospital ward staff notes often included a description of the physician’s thought process (e.g., phrases such as “I think” and “I feel”), which was largely absent from trainee notes across all hospitals.

#### Summary of events

In three of the 32 patient charts, no problem lists were documented for at least 48 hours after transfer from the ICU. Delays in addressing items in the problem list were also observed. For example, in the case of patient 030 (Hospital D), neurology consultants documented that they would assess the patient once an electromyogram (EMG) was completed. The EMG was deferred over the patient’s 7-day ward stay (Additional file 5: Table S5), resulting in a lack of consultation for this patient’s neurological issues.

### Structure

Analyses of the structure of physician progress notes largely examined whether information was presented in a standardized or non-standardized manner. Two subthemes emerged in the analysis of note structure: order of information, and style and accessibility of notes.

#### Order of information

Staff physician notes (ICU and ward) across all hospitals were structured in a non-standardized format, but had broad similarities in the sequence of information documented (i.e., patient history, exam(s)/interventions completed, and clinical plan). Trainee notes in nine of the ICUs and many most hospital wards followed a more standardized format that resembled the SOAP note structure [31]. One exception was that across all hospitals, surgical specialty notes were less structured and contained less detailed information than other notes in the sample (regardless of level of seniority) (Additional file 5: Table S5).

#### Style and accessibility

Generally, printed handwritten notes were more legible than cursive handwritten notes. Accessing sequential events in physician progress notes from the ICU that used an electronic template (Hospital H) was difficult as the typed template was on a separate piece of paper, inserted amongst pieces of paper with sequential handwritten notes, leading to non-chronologically ordered notes. ICU notes were typically documented in paragraph form whereas ward notes were largely documented in bullet point form with few words per line in nine of the ten hospitals. Notes from all hospitals included occurrences of questions posed by consulting physicians visiting the ICU that had been answered in previous notes (Additional file 5: Table S5).

### Purpose

The perceived purpose of physician progress notes (i.e., documentation of patient course) emerged across three main categories: preserving the patient’s story, decision-making, and documentation of communication.

#### Preserving the patient’s story

Across all hospitals, ICU notes and general medical teaching units regularly retained important elements of the ‘patient’s story’ (i.e., information reporting patient status, relevant medical or social history, patterns that emerged during care, and future-oriented care plan) [42, 43]. After transfer from ICU, the narrative of the patient’s story changed as notes were shorter and less structured with contextual factors (e.g., relevant history) commonly absent. The lack of continuity regarding the patient’s story was a key observation during the transition of care from ICU to hospital ward. For example, patient C-023’s ICU note details a “complicated hospitalization course” following ventral hernia repair that included multiple cardiac arrests and cardiac catheterizations, but in contrast, a ward note reported on wound and staple removal and the need for a cardiology follow-up (Additional file 5: Table S5).

Details of future-oriented care planning (e.g., rehabilitation needs) were more frequently documented in ward notes than in ICU notes across all sites. In Hospital H, a designated goals of care (i.e., resuscitative versus medical versus comfort) section was auto-populated. Goals of care designations were documented in nine of 32 medical records (including those from Hospital H), usually around the day of transfer, and specifically for patients with recently changed goals of care, or for those that had a recent meeting regarding prognosis.

#### Documentation of decision-making

Both ICU and ward notes routinely documented clinical plans, but rarely provided a decision-making rationale for plans requiring further justification (e.g., medications started and stopped, and clinical interventions applied without explanation or outcomes) (Additional file 5: Table S5). For example, in the chart of patient 001 (Hospital A), “family meeting to discuss prognosis…goals of care” was documented, however, no follow-up note was added to describe the nature or outcome of this conversation. Absence of decision-making documentation was a key observation during the transition of care from ICU to hospital ward.

#### Documentation of communication

The quantitative analyses showed that communication between providers or between providers and patients/family members consisted of a small portion of physician progress notes. When communication was documented, it most often appeared as directed provider-provider communication (Additional file 5: Table S5). However, documented responses to directed provider-provider communication were rare, resulting in many instances of incomplete textual information exchange (Additional file 5: Table S5).

## Discussion

Our study provides a comprehensive description of text-based communication during the transition of patient care between the ICU and hospital ward. Three key observations emerged from our analyses:

1. *Discrepancies in the documentation of patient problems*
  Observable differences were present in the problem lists documented by ICU and ward physicians in patient medical records including a lack of consistency between the last ICU note and first ward note. Structure and stylistic differences in the problem lists may impact continuity of patient care. For example, if care teams are used to sending and receiving information in different ways (e.g., head-to-toe assessment versus disease focused approach), there is potential for loss of important patient information at the point of patient transfer due to the incompatibility of preferred forms of communication. Differences in the continuity of patient problems at the point of transfer may indicate that previous notes are not being consistently reviewed by physicians. Barring verbal exchanges to fill gaps in patient information, there is a risk of information loss from the one consistent form of durable patient information available at the point of transfer. One strategy to address this challenge is to adapt the concept of medication reconciliation [44] for patient problems at times of transitions of care. Implementation of medication reconciliation systems has greatly reduced error rates and changes of medication orders [45]. Applying this approach to the handover of patient problems (e.g., by developing a comprehensive prioritized list of problems during a period of patient care, and describing whether the problems are active, resolved and/or in need of future action at the point of patient transfer) could pose as a potential solution for reducing the risk of information loss and the potential impact this has on patient care and outcomes at the point of transfer. EHRs could facilitate such a strategy by tracking problems over time.

2. *Lack of continuity regarding the patient’s story*
  Our results indicate that continuity of “patient stories” [42, 43] was often not maintained during transitions of care. Variation in the focus and purpose of written documentation between ICUs and hospital wards were identified as key barriers to maintaining continuity in the patient’s story. In most cases, after transfer from ICU, less detail was used to describe patient history and contextual factors that may have been implicated in the patient’s initial admission to hospital. Consequently, patients who have ongoing issues that are outside the clinical scope of focus of the physicians assuming care may be at increased risk of these issues not being attended to [5, 6]. Providing patients and families with concurrent access to their medical record [46] could potentially decrease information loss across care settings and empower patients and families to function as a source of continuity in maintaining the patient’s story [47]. In addition, a standardized communication tool that synthesizes important patient information by documenting recurrent themes and forcing record keeping of essential aspects of care (e.g., goals of care) is needed to safeguard against information loss during transitions of care.

3. *Absence of documentation in decision-making*
  Rationale for clinical decisions was rarely documented in physician progress notes (i.e., *how* and *why* decisions were made), which may be attributed to workload and time available, or an attempt to streamline textual documentation [22]. The absence of documented rationale for clinical decisions represents an important communication gap since ICU patients routinely receive care from many different providers and transitions of care are common. One strategy to address this gap would be to add a ‘why’ subheading to the ‘Plan’ portion of the SOAP model (i.e., SOAPy) to prompt note writers to document the rationale for the plan selected. EHRs could be effective tools for prompting rationale documentation.

Furthermore, while EHRs have been shown to improve accuracy and readability of progress notes through the automatic population of routine information (e.g., date, time, signature) and the removal of penmanship errors [36] recent literature has demonstrated that this does not necessarily result in an effective capture of the patient’s story [43, 48]. Furthermore, our data show that clinical impressions and documentation of communication were more difficult to identify at the ICU site that utilized electronic notes due to the rigid structure and amount of information presented [36, 49]. Accessibility of retrieving and documenting communication, clinical impression, and the patient’s story remains an important issue that should be addressed in the design and development of any standardized communication tool (handwritten or electronic) that is utilized during transitions of care.

### Limitations

Our study findings have some limitations. First, although common across Canada, the hospitals included exclusively used paper-based medical records for physician progress notes, and therefore it is unclear how our results would have been influenced by the inclusion of EHR notes. The discrepancies in documentation of problem lists, decision-making and continuity of the patient story are unlikely to be specific to paper-based records. Second, for feasibility reasons, content analyses were performed on English-language medical records. Third, we only examined notes that were written by physicians. Future research should examine allied health professional documentation for communication.

## Conclusions

Documentation of patient problems, decision-making, and the patient’s story, emerged as central points of discontinuity for patients transferred from ICU to hospital ward. This discontinuity can result in the loss of important information during transitions of patient care and ultimately impact the care patients receive. Key strategies for improving information loss in the context of varied approaches to textual documentation (including strategies targeted at retaining a thick description of patient status, relevant medical and social history, patterns that emerged during care, and future-oriented care plan) should be implemented. Moreover, future research should investigate the implications of different approaches to note writing on patient outcomes (i.e., incidence of adverse events) in an effort to define best practice. Defining standards for the design and development of both handwritten and electronic progress notes is a top priority for future research.

## Additional files

Additional file 1: Number of patient medical records collected per study site. Total number of patient medical records and number of physician progress notes per study site included in this analysis. (DOC 31 kb)

Additional file 2: Legend of patient and site identifiers. Patient identifiers corresponding to the examples in the content analyses, distinguished by site and type of patient (surgical versus non-surgical). (DOC 31 kb)

Additional file 3: Quantitative descriptive analysis of physician progress notes for medical patients. Physician progress notes for medical patients categorized according to patient location during the 10-day period. (DOC 45 kb)

Additional file 4: Quantitative descriptive analysis of physician progress notes for surgical patients. Physician progress notes for surgical patients categorized according to patient location during the 10-day period. (DOC 45 kb)

Additional file 5: Themes, subthemes and examples from content analysis. Textual examples for each theme and subtheme from the content analysis. (DOC 978 kb)

## Abbreviations

EHRs: Electronic health records
EMG: Electromyogram
ICU: Intensive care unit
REB: Research Ethics Board
SOAP: Subjective objective, assessment, and plan
