# Supplementary material for: Transfers from intensive care unit to hospital ward: a multicentre textual analysis of physician progress notes
Source: Crit Care. 2018 Jan 28;22:19. doi: 10.1186/s13054-018-1941-0 (PMC5787341; doi:10.1186/s13054-018-1941-0)
Supplement: Supplementary file 1 — Number of patient medical records collected per study site. Total number of patient medical records and number of physician progress notes per study site included in this analysis. (DOC 31 kb) [file 13054_2018_1941_MOESM1_ESM.doc]

| **Table S1.** Number of Patient Medical Records Collected Per Study Site | | |
| --- | --- | --- |
| **Study Site** | **Number of Medical Records** | **Number of Notes** |
| Site A | 24 | 628 |
| Site B | 50 | 555 |
| Site C | 33 | 482 |
| Site D | 26 | 344 |
| Site E | 48 | 1,005 |
| Site F | 54 | 724 |
| Site G | 77 | 1,211 |
| Site H | 58 | 943 |
| Site I | 43 | 686 |
| Site J | 34 | 623 |
| **Total** | **447** | **7,201** |
