# Supplementary material for: Transfers from intensive care unit to hospital ward: a multicentre textual analysis of physician progress notes
Source: Crit Care. 2018 Jan 28;22:19. doi: 10.1186/s13054-018-1941-0 (PMC5787341; doi:10.1186/s13054-018-1941-0)
Supplement: Supplementary file 2 — Legend of patient and site identifiers. Patient identifiers corresponding to the examples in the content analyses, distinguished by site and type of patient (surgical versus non-surgical). (DOC 31 kb) [file 13054_2018_1941_MOESM2_ESM.doc]

| **Table S2.** Legend of Patient and Site Identifiers | | |
| --- | --- | --- |
| **Site** | **Patient ID** | **Type of Patient** |
| A | 001  002  003  004 | Non-Surgical  Non-Surgical  Surgical  Surgical |
| B | 010  011  012  013 | Non-Surgical  Non-Surgical  Surgical  Surgical |
| C | 020  021  022  023 | Non-Surgical  Non-Surgical  Surgical  Surgical |
| D | 030  031  032  033 | Non-Surgical  Non-Surgical  Surgical  Surgical |
| E | 040  041  042  043 | Non-Surgical  Non-Surgical  Surgical  Surgical |
| F | 050  051  052  053 | Non-surgical  Non-surgical  Surgical  Surgical |
| G | 060  061  062  063 | Non-Surgical  Non-Surgical  Surgical  Surgical |
| H | 070  071  072  073 | Non-Surgical  Non-Surgical  Surgical  Surgical |
