# Supplementary material for: Transfers from intensive care unit to hospital ward: a multicentre textual analysis of physician progress notes
Source: Crit Care. 2018 Jan 28;22:19. doi: 10.1186/s13054-018-1941-0 (PMC5787341; doi:10.1186/s13054-018-1941-0)
Supplement: Supplementary file 3 — Quantitative descriptive analysis of physician progress notes for medical patients. Physician progress notes for medical patients categorized according to patient location during the 10-day period. (DOC 45 kb) [file 13054_2018_1941_MOESM3_ESM.doc]

**Table S3.** Quantitative Descriptive Analysis of Physician Progress Notes for Medical Patients

| **Measures** | **Total**  **(n=4,357)** | **ICU Stay**  **(n=1,158)** | **Transfer Day**  **(n=777)** | **Ward Stay**  **(n=2,422)** | **p-value*^a^*** |
| --- | --- | --- | --- | --- | --- |
| Mean Number of Notes Per Day (Per Patient) | 2.1  [1.9-2.3] | 2.3  [2.0-2.6] | 2.9  [2.6-3.1] | 1.8  [1.6-2.1] | p=0.002 |
| Handwritten | 97%  [92%-100%] | 92%  [85%-100%] | 93%  [86%-100%] | 100%  [93%-100%] | p<0.001 |
| Legible | 88%  [80%-95%] | 87%  [79%-95%] | 88%  [80%-96%] | 88%  [81%-96%] | p=0.551 |
| Date Included | 95%  [92%-97%] | 94%  [92%-97%] | 96%  [93%-99%] | 95%  [93%-98%] | p=0.543 |
| Time-Stamped | 50%  [42%-59%] | 50%  [41%-60%] | 55%  [45%-64%] | 48%  [39%-57%] | p=0.406 |
| Signature/Name | 49%  [35%-63%] | 48%  [35%-62%] | 52%  [38%-65%] | 49%  [35%-62%] | p=0.896 |
| Mean Number of Lines Per Note (Per Patient) | 18.8  [17.0-20.7] | 22.2  [20.0-24.4] | 21.0  [18.7-23.2] | 16.7  [14.4-18.9] | p<0.001 |
| Patient History*^b^* | 10%  [7%-13%] | 11%  [8%-14%] | 11%  [8%-14%] | 8%  [5%-11%] | p<0.001 |
| Patient Data*^b^* | 58%  [52%-64%] | 56%  [51%-62%] | 57%  [51%-63%] | 60%  [54%-65%] | p=0.015 |
| Patient Care Plan*^b^* | 32%  [26%-38%] | 32%  [26%-39%] | 32%  [26%-39%] | 33%  [26%-39%] | p=0.962 |
| Communication*^c^* | 10%  [8%-12%] | 8%  [5%-10%] | 11%  [9%-14%] | 11%  [8%-13%] | p=0.039 |
| Provider-Provider*^d^* | 56%  [49%-63%] | 66%  [56%-77%] | 63%  [52%-73%] | 52%  [44%-60%] | p=0.019 |
| Provider-Family*^d^* | 21%  [14%-28%] | 20%  [10%-29%] | 22%  [12%-32%] | 22%  [14%-30%] | p=0.606 |
| Provider-Patient*^d^* | 39%  [32%-46%] | 27%  [15%-38%] | 37%  [26%-49%] | 43%  [33%-52%] | p=0.009 |
| Data are presented as mean proportions (95% confidence intervals) unless otherwise indicated. Data include notes written by ICU and Ward physicians categorized according to patient location during the ten day period. Indented variables are presented as distributions.  *^a^* P-values represent the comparison between the ICU stay and ward stay for each variable.  *^b^* Patient history (e.g., symptoms), data (e.g., laboratory results), and care plan (e.g., treatments prescribed) are presented as mean proportions (%) of the total number of lines for each note.  *^c^* Documentation of any communication between providers, between providers and family members, and between providers and patients.  *^d^* Provider-provider, -family, -patient communication presented as mean proportions (%) of the total documented communication that is not mutually exclusive. | | | | | |
