# Supplementary material for: Transfers from intensive care unit to hospital ward: a multicentre textual analysis of physician progress notes
Source: Crit Care. 2018 Jan 28;22:19. doi: 10.1186/s13054-018-1941-0 (PMC5787341; doi:10.1186/s13054-018-1941-0)
Supplement: Supplementary file 4 — Quantitative descriptive analysis of physician progress notes for surgical patients. Physician progress notes for surgical patients categorized according to patient location during the 10-day period. (DOC 45 kb) [file 13054_2018_1941_MOESM4_ESM.doc]

**Table S4.** Quantitative Descriptive Analysis of Physician Progress Notes for Surgical Patients

| **Measures** | **Total**  **(n=2,695)** | **ICU Stay**  **(n=808)** | **Transfer Day**  **(n=430)** | **Ward Stay**  **(n=1,457)** | **p-value*^a^*** |
| --- | --- | --- | --- | --- | --- |
| Mean Number of Notes Per Day (Per Patient) | 2.1  [1.9-2.3] | 2.6  [2.3-2.9] | 2.6  [2.3-2.9] | 1.7  [1.4-2.0] | p<0.001 |
| Handwritten | 97%  [93%-100%] | 94%  [88%-100%] | 93%  [87%-99%] | 100%  [96%-100%] | p<0.001 |
| Legible | 86%  [75%-97%] | 84%  [74%-95%] | 86%  [75%-97%] | 86%  [75%-97%] | p=0.644 |
| Date Included | 95%  [91%-99%] | 95%  [91%-100%] | 96%  [92%-100%] | 95%  [90%-99%] | p=0.772 |
| Time-Stamped | 47%  [36%-58%] | 53%  [41%-65%] | 52%  [30%-64%] | 42%  [30%-54%] | p<0.001 |
| Signature/Name | 48%  [36%-61%] | 50%  [35%-64%] | 46%  [32%-61%] | 46%  [31%-60%] | p=0.264 |
| Mean Number of Lines Per Note (Per Patient) | 15.3  [13.7-16.9] | 19.2  [17.2-21.3] | 17.2  [15.2-19.3] | 12.1  [10.0-14.2] | p<0.001 |
| Patient History*^b^* | 10%  [7%-12%] | 11%  [8%-14%] | 10%  [8%-13%] | 7%  [4%-9%] | p<0.001 |
| Patient Data*^b^* | 62%  [58%-66%] | 60%  [55%-64%] | 60%  [55%-64%] | 65%  [61%-70%] | p=0.001 |
| Patient Care Plan*^b^* | 29%  [22%-33%] | 30%  [25%-35%] | 30%  [25%-35%] | 28%  [24%-33%] | p=0.365 |
| Communication*^c^* | 7%  [5 %-9%] | 7%  [4%-9%] | 4%  [2%-6%] | 6%  [4%-9%] | p=0.916 |
| Provider-Provider*^d^* | 57%  [46%-68%] | 62%  [46%-78%] | 61%  [38%-83%] | 52%  [38%-66%] | p=0.282 |
| Provider-Family*^d^* | 25%  [15%-35%] | 28%  [16%-41%] | 19%  [0%-38%] | 21%  [10%-32%] | p=0.332 |
| Provider-Patient*^d^* | 34%  [23%-45%] | 31%  [17%-46%] | 31%  [10%-53%] | 39%  [26%-52%] | p=0.356 |
| Data are presented as mean proportions (95% confidence intervals) unless otherwise indicated. Data include notes written by ICU and Ward physicians categorized according to patient location during the ten day period. Indented variables are presented as distributions.  *^a^* P-values represent the comparison between the ICU stay and ward stay for each variable.  *^b^* Patient history (e.g., symptoms), data (e.g., laboratory results), and care plan (e.g., treatments prescribed) are presented as mean proportions (%) of the total number of lines for each note.  *^c^* Documentation of communication between providers, between providers and family members, and between providers and patients.  *^d^* Provider-provider, -family, -patient communication presented as mean proportions (%) of the total documented communication that is not mutually exclusive. | | | | | |
