# Supplementary material for: Transfers from intensive care unit to hospital ward: a multicentre textual analysis of physician progress notes
Source: Crit Care. 2018 Jan 28;22:19. doi: 10.1186/s13054-018-1941-0 (PMC5787341; doi:10.1186/s13054-018-1941-0)
Supplement: Supplementary file 5 — Themes, subthemes and examples from content analysis. Textual examples for each theme and subtheme from the content analysis. (DOC 978 kb) [file 13054_2018_1941_MOESM5_ESM.doc]

| **Table S5.** Themes, Sub-Themes and Examples from Content Analysis | | | | |  | |
| --- | --- | --- | --- | --- | --- | --- |
| **Themes** | **Sub-Themes** | **Examples** | | | | |
|  |  | **Site-Patient ID** | **Data** | | | **Interpretation** |
| Focus | Problem List | Site G - 062 | 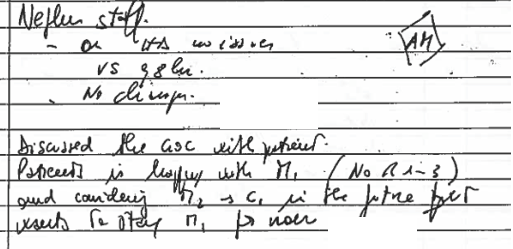 | 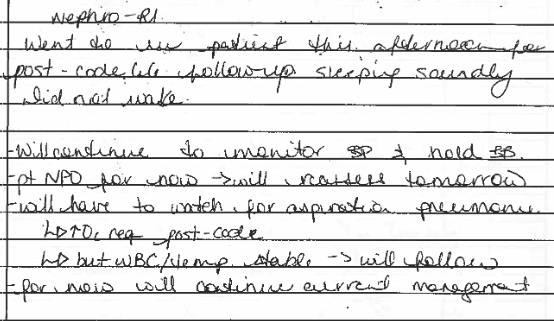 | | Demonstrates the difference in problem list between staff (left image) and trainee (right image) notes. Problems identified in the staff note focuses on communication and GOC, while the trainee note focuses on clinical orders. |
|  |  |  |  |  |  |  |
|  | Clinical impression | Site C - 022 | 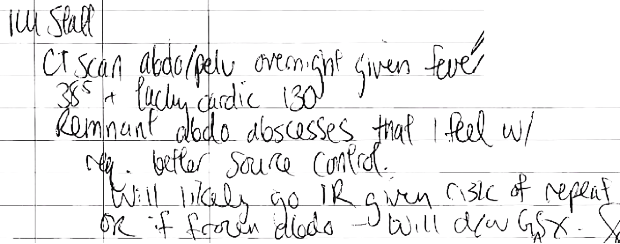 | | | Demonstrates staff notes often contain thought process and phrases such as “I feel…”. |
|  | Summary of Events | Site D - 030 | August 12: ICU - “needs neuro consult”  August 14: Ward - “neuro will re-see [pt] after EMG”  August 15: Ward - “EMG + NCS ordered”  August 17: Ward - “EMG will be done this week” and separate note “[follow-up] by neuro…+EMG ordered”  August 18: Ward - “still waiting EMG, then [follow-up] by neuro” and separate note by another specialty - “I will perform EMG/NCS during my EMG clinic on Thursday, Aug 20” | | | Delay in addressing items in the problem list during handover process, continued delay throughout ward stay. |
| Structure | Order of Information | Site E - 043 | 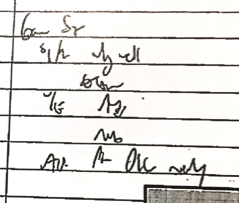 | 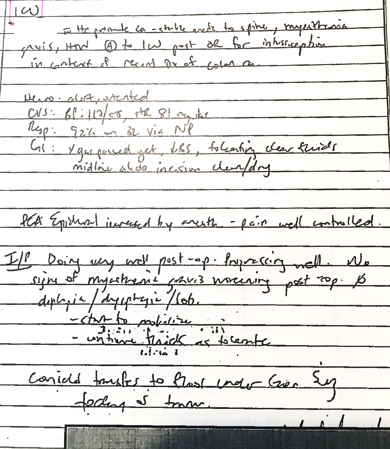 | | The notes from surgical specialties (left image) had less structure and included less detailed information compared to typical ICU notes (right image). |
|  | Note Style and Information Accessibility | Site B - 010 | 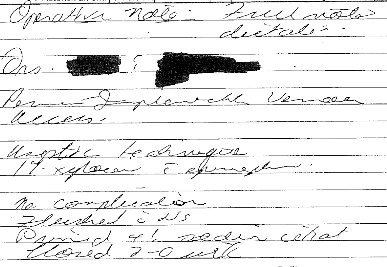 | | | Image presented demonstrates cursive handwriting style often leads to more illegible notes. |
|  |  | Site G - 061 | Trauma consult in ICU. Trauma physician notes they are happy to accept care, then lists several questions in progress notes that have already been answered in previous notes on numerous occasions (e.g. “No-op as per plastics?”). When information is clearly documented in a patient’s chart, other care providers are not necessarily reviewing this information or was unable to locate information. | | | Differences in written style and reviewing previous notes. |
|  |  |  |  |  |  |  |
| Purpose | Preserving the Patient’s-Story | Site C - 023 | 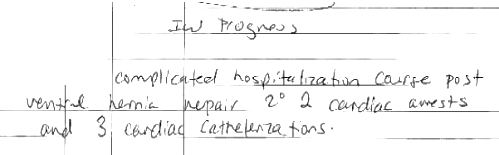 | 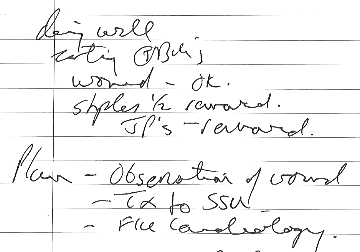 | | Demonstrates the lack of patient story between notes, ICU note (left) and ward note (right). |
|  | Decision-Making | Site G - 060 | Day 2  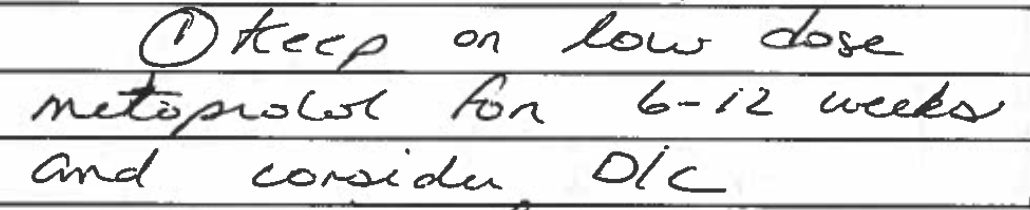  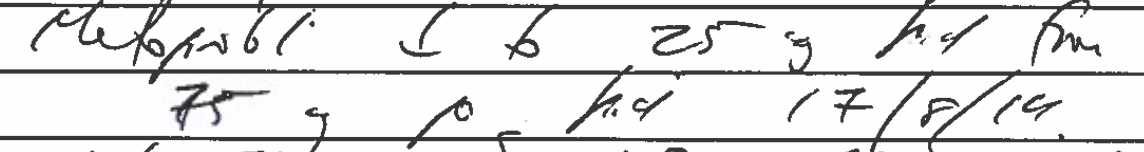  Day1 | Day 3  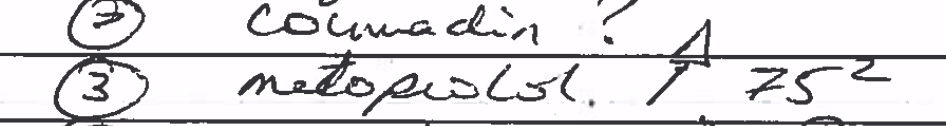 | | Patient’s dose of metroprolol was decreased on Day 1, Day 2 notes confirms and reiterates the low dose. However, on Day 3 the dose increased again with no rationale. |
|  | Documentation of Communication | Site G - 062 | 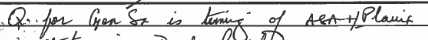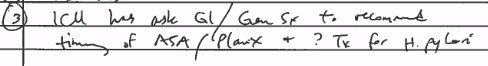 | | | ICU physician (top image) asked a question to “Gen Sx”. Nephrology (bottom image) reiterated the question. “Gen Sx” or “GI” did not document a note communicating the answer. |
|  |  | Site C - 020 | 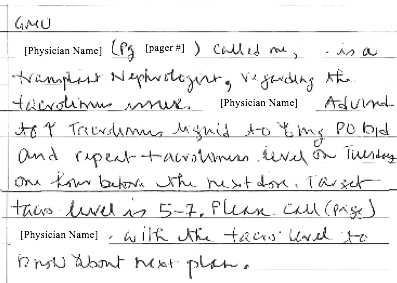 | | | Ward physician documenting a phone conversation. |
